# Epigenetic Application of ATAC-Seq Based on Tn5 Transposase Purification Technology

**DOI:** 10.1155/2022/8429207

**Published:** 2022-08-11

**Authors:** Wangchun Li, U Tim Wu, Yu Cheng, Yanhao Huang, Lipeng Mao, Menghan Sun, Congling Qiu, Lin Zhou, Lijuan Gao

**Affiliations:** ^1^Department of Critical Care Medicine, Shunde Hospital Affiliated of Jinan University, 528305 Foshan, China; ^2^Meng Yi Center Limited, Macau 999078, China; ^3^Institute of Geriatric Immunology, School of Medicine, Jinan University, 510632 Guangzhou, China; ^4^Department of Microbiology and Immunology, School of Medicine, Jinan University, Guangzhou, China; ^5^Nanjing Maternity and Child Health Care Hospital, Women's Hospital of Nanjing Medical University, No. 123, Tianfeixiang, Mochou Rd., Nanjing 210004, China

## Abstract

**Background:**

Assays of transposase accessible chromatin sequencing (ATAC-seq) is an efficient assay to investigate chromatin accessibility, which depends on the activity of a robust Tn5 transposase to fragment the genome while cutting in the sequencing adapters.

**Methods:**

We propose reliable approaches for purifying hyperactive Tn5 transposase by chitin magnetic bead sorting. Double-stranded DNA of J76 cells and 293T cells were digested and subjected to tagmentation as test samples with Tn5 transposase, and libraries were established and sequenced. Sequencing data was then analyzed for peak calling, GO enrichment, and motif analysis.

**Results:**

We report a set of rapid, efficient, and low-cost methods for ATAC-seq library construction and data analysis, through large-scale and rapid sequencing. These methods can provide a reference for the study of epigenetic regulation of gene expression.

## 1. Introduction

In mammalian and other eukaryotic cells, the nuclear DNA encoding genetic information winds around the histone to form nucleosomes, further folds and condenses to form chromatin [1]. Transcriptionally active euchromatin is in a loose and open state that is accessible and consists of transcriptionally active genes and various cis-regulatory elements. Euchromatin may exhibit direct epigenetic changes such as DNA transcription, methylation, and histone modification [2, 3]. In addition, different stages of biological development, distinct types of cells, and different physiological or environmental conditions will affect the changes in chromatin accessibility to regulate gene expression. Therefore, chromatin accessibility is essential to the epigenetic regulation of gene expression [4, 5]. With the rapid development of high-throughput sequencing analysis, there are multiple assays to facilitate chromatin accessibility research, including assays of transposase accessible chromatin sequencing (ATAC-seq), DNase I hypersensitive site sequencing (DNase-seq), and formaldehyde-assisted isolation of regulatory elements sequencing (FAIRE-seq) [6–8]. The study of chromatin accessibility has many applications for revealing many biological questions, such as identifying transcription factor binding sites, mapping nucleosomes, investigating the differential activity of DNA regulatory elements, and carrying out epigenetic research [9]. ATAC-seq is a highly efficient approach to studying chromatin accessibility.

Nevertheless, the activity of a hyperactive Tn5 transposase is essential to ATAC-seq. This transposase works through tagmentation, which fragments the genome while inserting the sequencing adapter. These sequences can be amplified by PCR and sequenced, which was typically performed using 2–4 orders of magnitude fewer cells than other chromatin accessibility sequencing methods, and fewer experimental procedures [10].

Therefore, Tn5 transposase has recently become a research hotspot, and its production technology has been continuously optimized and improved. The mutation site of wild-type Tn5 is M56, and the transposition frequency is quite low. After introducing the mutants of E54K and L372P, Tn5 transposase is hyperactive for tagmentation [11–13]. In the downstream purification process of Tn5 transposase, chitin purification columns are mainly used. This complicated method is laborious and cannot be reliably reproduced in laboratories [14].

Here, we experimented with a simple procedure for fast producing tagmentation-efficient Tn5 transposases, and all the main steps for efficient generation of a sequencing library are depicted without changing the hyperactivity of Tn5 transposase [15, 16]. To detect the activity of the purified Tn5 transposase and establish a complete and efficient ATAC-seq library and sequencing method, the metabolic system representative human kidney epithelial cell line 293T and human peripheral blood leukemia T cell line J76 were chosen as test samples for the ATAC-seq library construction. Then the data were analyzed using methods of systems biology. The difference in the peaks of open chromatin in the two cell lines was compared, and the information of open chromatin location, nucleosome location, and transcription factor binding sites was obtained. The regulatory regions of functional genes were identified, and potential binding proteins were predicted. The changes of chromatin open regions in different periods or states were compared with advanced epigenetic analysis. Our study provides an improved methodology for mass producing hyperactive Tn5 transposase for reliable ATAC-seq analysis.

## 2. Materials and Methods

### 2.1. Materials and Reagents

#### 2.1.1. Strains, Plasmids, and Cells

HEK293T cells (human embryonic kidney cells) and Jurkat cells (human acute T-lymphoblastic leukemia cells, J76 cells) were kindly provided by Dr. Hongyi Zhang of Jinan University, China. Ptxb1-Tn5 were purchased from Addgene, and BL21 cells were preserved in our laboratory.

#### 2.1.2. Main Reagents and Instruments

A PCR Purification Kit (Omega) and chitin magnetic beads were purchased from Biolabs. Dialysis bags were purchased from Solarbio Company in Beijing, and primers were synthesized by Biotechnology (Shanghai) Co., Ltd. Other instruments include centrifuge (Eppendorf), Thermal Cycler (Hangzhou Langji), and Protein concentration analyzer (Quawell).

### 2.2. Methods

#### 2.2.1. Tn5 Production and Purification

Take out 8 *µ*·l Ptxb1-Tn5 recombinant plasmid and transform it into 100 *µ*·l BL21 competent cells. Transformed cells were added to 1 ml·LB culture medium and incubated overnight at 37°*µ*·C. When the OD of the culture reached 0.5–0.7, it was expanded to 400 ml of culture with 100 *µ*·g/ml ampicillin to reach an OD = 0.9, then added to 250 *µ*·l of 1 M IPTG for 4 h at 23°C. When the culture reached A600 = 3.0, the precipitates were collected by centrifugation (4°C, 5000 × *g*, 15 min), the supernatant was removed, and the pellets were frozen at −80°C overnight. After adding HEGX (20 mM HEPES, 0.8 M NaCl, 1 mM EDTA, 10% glycerol, 0.2% Triton X-100, pH = 7.2) to the pellets, ultrasonic cracking was performed. Lysis was carried out with 11 cycles of 47 bursts with a 30% duty cycle at output 7 on a sonicator with intermittent cooling on ice in a beaker. Sediment was removed by centrifugation at 14,000 × *g* for 30 min at 4°C. Add 10% neutralized PEI dropwise to the supernatant and stir with magnetic force. To the supernatant, 10% neutralized PEI was added dropwise on a magnetic stirrer, and then the lysed precipitate was removed by centrifugation at 15000 × *g* for 10 min at 4°C. *E*. *coli* genomic DNA was precipitated by adding 10% PEI.

The supernatant was mixed with chitin magnetic beads and then suspended in cold HEGX buffer. These beads were previously washed in 100 ml of HEGX buffer and bonded on the rotator for 4 hours at 4°C. The beads were collected using magnetic support, incubated with HEGX buffer for 5 minutes at 4°C, and washed 3 times. Lastly, the beads were resuspended in cold HEGX buffer with 100 mm DTT and cultured on a rotator at 4°C for 36–48 h. In this process, the fusion protein was cut from the intein-CBD domain and pure Tn5 was dissolved into the solution. Collect the beads again with magnetic support and transfer the supernatant to a new tube. The magnetic beads were washed three times with cold HEGX buffer, the eluent of each cleaning was transferred to a new tube, and the eluent containing protein was combined. The elution was dialyzed by using dialysis buffer (Dialysis Buffer, DF, 2 X Tn5 DF, 100 mM Hepes, pH 7.2, 200 mM NaCl, 2 mM DTT, 0.2 mM EDTA, 20% glycerol, 0.2% Triton X-100) in a dialysis bag. The concentration of Tn5 was measured by a Nanodrop spectrophotometer. If the absorbance is A280 = 3.0 or higher, it is directly used for transposome assembly. The Tn5 solution was stored in a solution containing 55% glycerin (100% glycol: 2 x Tn5 DB : Tn5 protein extraction solution volume ratio was 55 : 16.5 : 28.5).

#### 2.2.2. Assay of Transposase Activity

Each time a new batch of the enzyme was purified, an assay of its activity should be performed. This can be skipped if an old batch with known activity is used. The reaction system was 14 *µ*·l H_2_O, 1 *µ*·l 50 ng/ml DNA, 4 *µ*·l 5 x TAPS-MgCl_2_-PEG 8000 (50 mM TAPS-NaOH, pH = 8.5, 25 mM MgCl2, 40% PEG 8000), and 1 *µ*·l 3.4 *µ*·g/*µ*·l Tn5 enzyme. Four reaction systems were set up according to the concentration of the Tn5 enzyme. The reaction was carried out on a thermal circulator with a cover temperature of 95°C and was incubated at 55°C for 10 minutes. The reaction was terminated by adding 2.5 *µ*·l 0.2% SDS (final concentration 0.02%) to each reaction and incubated at 55°C for 7 minutes. The samples were then analyzed by agarose gel electrophoresis.

#### 2.2.3. Tn5 Transposome Assembly

For transposome assembly, the preannealed mosaic end double-stranded (MEDS) oligonucleotides can be operated in solution as follows:

  Tn5 ME-A: 5′-TCGTCGGCAGCGTCAGATGTGTATAAGAGACAG-3′;
  Tn5 ME-B: 5′-GTCTCGTGGGCTCGGAGATGTGTATAAGAGACAG-3′;
  Tn5 MErev: 5′-[phos] CTGTCTCTTATACACATCT-3′.

The underlined region corresponds to the double-stranded portion of the adapter-ME sequence identified by the transposase. An equimolar mixture of Tn5 ME-A and Tn5 ME-B was annealed and connected to Tn5 MErev to generate the adapters Primer A and Primer B. Tn5 was assembled with 128 *µ*·l 2 x DF, 64 *µ*·l Primer A, 64 *µ*·l Primer B, 100 *µ*·l 3.4 *µ*·g/*µ*·l Tn5 transposase and incubated at room temperature for at least 1 h. We have extended the incubation time to several hours without problems.

#### 2.2.4. Tagmentation Reaction

To evaluate the self-made Tn5 transposomes, the DNA of samples was digested and subjected to fragmentation, and the library was created for ATAC-seq. The reaction system was as follows: 14 *µ*·l H_2_O, 1 *µ*·l 54 ng/ml DNA, 4 *µ*·l 5 x TAPS-MgCl_2_-PEG 8000, and 1 *µ*·l 0.96 *µ*·g/*µ*·l in-house Tn5 Transposase. The reaction conditions were the same as 2.2.2.

#### 2.2.5. Cell Culture

293T cells preserved in liquid nitrogen were thawed rapidly in a water bath at 37°C, centrifuged for 5 min, and then the supernatant was discarded. A DMEM culture medium including 1% penicillin-streptomycin and 10% fetal bovine serum was added, and cells were cultured in an incubator with 5% CO_2_ and a saturated humidity of 37°C. When the cell density achieved 85%–90%, cells were rinsed twice with PBS, trypsinized at 37°C for 1–2 min, and after complete cell detachment, the medium was added to stop digestion. The cultured cells were centrifuged at 600 x g for 5 min. The supernatant was removed; the cells were recultured by adding DMEM medium. J76 cells preserved in liquid nitrogen were thawed rapidly in a 37°C water bath, centrifuged for 5 min, and the supernatant was discarded. The cells were cultured with RPMI1640 medium containing 10% fetal bovine serum in an incubator with 5% CO_2_ and a saturated humidity of 37°C. The culture medium was replaced every 48 hours. When the cell density was achieved at 80% to 90%, the cells were centrifuged, rinsed twice with PBS, and then RPMI1640 medium was added and the cells were returned to culture.

#### 2.2.6. Tagmentation-Based ATAC-Seq Library Preparation Using In-House-Produced Tn5

The numbers of 293T cells and J76 cells in the logarithmic growth phase were counted by hemacytometer. Fifty thousand cells were added to PCR tubes and centrifuged at 4°C at 600 × *g* for 5 min, then the supernatant was discarded. The precooled 0.1% NP-40 lysis buffer solution was added to the cell precipitate and pipetted until homogeneous. The cells were lysed on ice for 3 minutes. After centrifugation at 4°C 550 × *g* for 5 min, discard the supernatant. The two kinds of DNA samples were cut by adding the self-made Tn5 transposase mixed system into the precipitation, and the library was constructed before ATAC-seq sequencing. The reaction system was 10 *µ*·l 5 x TAPS-MgCl2-PEG 8000, 32 *µ*·l H2O, 8 *µ*·l 0.96 *µ*·g/*µ*·l Tn5 enzyme. The reaction conditions were the same as for 1.2.3 enzyme activity detection.

The DNA of Tn5 reaction system samples was purified by an Omega-PCR purification kit. The purified DNA samples were collected and amplified by PCR. The DNA samples were sent for sequencing. A portion of the DNA was used for QPCR to measure the concentration of DNA.

### 2.3. Data Analysis

The original data of ATAC-seq were considered preanalysis. The experimental process of ATAC-seq analysis includes prealignment quality control (QC), read alignment to a reference genome, and postalignment QC and processing. The sequence was aligned back to the reference genome by Bowtie2 software and the repetitive sequences were removed by SAM tools. In order to facilitate reading and visualization, deepTools were used for visualization, MACS2 for peak calling, the data was converted into BAM file format, and then peak annotation, GO enrichment, and motif analysis were carried out, respectively [17, 18]. In the course of data analysis and processing, *t*-test was used to select the data that meet |lgfc| > 1.00 and *P* < 0.05 at the same time.

## 3. Results

### 3.1. Expression Process and Purification Procedure of Tn5 Transposase

**Figure 1:**
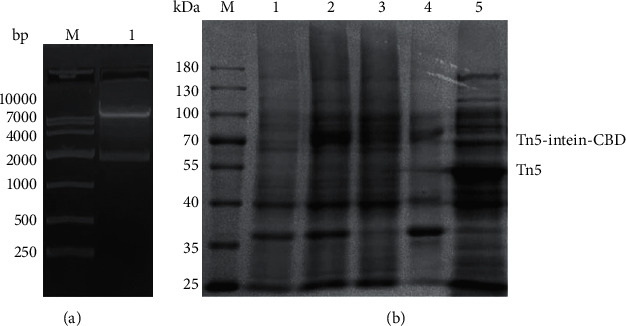
Expression and purification of Tn5 protein. (a) Ptxb1-Tn5 plasmid identification. M Marker; line 1: plasmid digested by Xbal/BamHI. (b) Expression and purification of Tn5 protein by SDS-PAGE electrophoresis, wild-type Tn5 and higher-molecular weight Tn5-intein-CBD is seen above. M Marker; line 1: crude lysate before IPTG induction; line 2: sonication supernatant; line 3: flow-through; line 4: elution after magnetic beads; line 5: DTT eluted fractions.

pTXB1-Tn5 with a C-terminal intein tag and a chitin-binding domain (CBD) was transformed into BL21 and expressed. Ptxb1-Tn5 plasmid identification is shown in Figure 1(a), where Tn5-intein was digested with XbaI/BamHI to obtain a product of about 2000 bp [12]. Expression of the Tn5 fusion protein was induced with IPTG and continued to culture until A600 = 3.0. The cells were lysed by ultrasound, the cell DNA in the lysate solution was discarded by polyethyleneimine (PEI) precipitation, and the lysate supernatant was loaded with chitin magnetic beads. The intein–CBD tag was cut by DTT, and Tn5 was released from the magnetic beads into the solution.

Tn5 in the process of production and purification was analyzed by SDS-PAGE gel electrophoresis (Figure 1(b)). Results showed that Tn5 protein was successfully expressed and high purity was obtained.

### 3.2. Prime Activity Assay of the Tn5 Transposase

**Figure 2:**
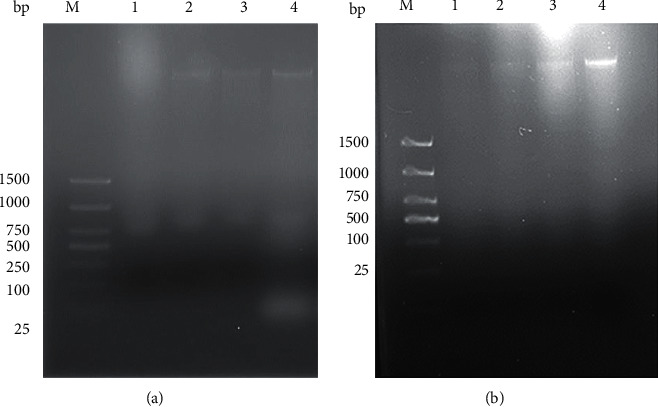
Activity tests and tagmentation demonstrated with the Tn5 transposase. (a) M: Marker; line 1–3: several reactions were performed simultaneously with different dilutions of Tn5 ranging from 1.25 ng/ml, 2.5 ng/ml, and 5 ng/ml; line 4: control group. (b) DNA sample digested by Tn5 transposase. M Marker; line 1–3: the volume of Tn5 enzyme added in different reactions was 8 *µ*·l, 6 *µ*·l, and 4 *µ*·l. Line 4: control group.

When a new batch of the enzyme is purified, confirmation of its activity should be performed. Several reactions were performed simultaneously using Tn5 diluents with different concentration gradients, from 1.25 to 5 ng/ml, while maintaining the gDNA concentration at 50 ng/ml (Figure 2(a)). Tn5 transposase can digest DNA into diffuse bands with 5 ng/ml Tn5. In general, the study shows that homemade Tn5 is active, which is suitable for the construction of a library with small amounts of samples.

### 3.3. Transposome Assembly and Tagmentation of DNA Sample Fragments

After the assembly of Tn5 transposase, the activity of the homemade Tn5 transposome assembly must be assessed. We carried out a few reactions concurrently with a different concentration gradient of Tn5 ranging from 4 *µ*·l, 6 *µ*·l, and 8 *µ*·l, while the gDNA concentration remained constant at 50 ng/mL. The reaction products after homemade Tn5 digestion were identified by agarose gel electrophoresis, illustrating the fragment length of sample DNA (Figure 2(b)). Tn5 transposome assembly can entirely digest DNA into diffuse bands with 8 ng/ml Tn5, and visualization of the input DNA can be performed via gel electrophoresis.

### 3.4. Tagmentation-Based ATAC-Seq Library Construction Procedure

In order to establish a tagmentation-based ATAC-seq library preparation protocol that is independent of the homemade assembled Tn5 transposase, genomic DNA extracted from 293T and J76 cells was dealt with Tn5 transposomes, which combined with DNA, embed breaks and ligates oligonucleotide adapters to the 5′- ends of fragmentation sites. The purified DNA samples were collected and amplified by PCR, then sequenced.

### 3.5. Quality Control (QC) of ATAC Sequencing Initial Data

Preanalysis and quality control of the sequenced data are necessary. After reading the trimming instructions, fast QC and trimming can be carried out again to verify that the adapter was successfully removed. BWA was used to filter and remove low-quality bases. And then trimmed reads were mapped to a related genome, taking advantage of Bowtie2. In brief, the procedures mentioned above enhance the capability of open chromatin detection and reduce the output of erroneous results. The results met the standard and the data quality was excellent.

### 3.6. Signal Enrichment around Transcription Start Sites (TSS)

**Figure 3:**
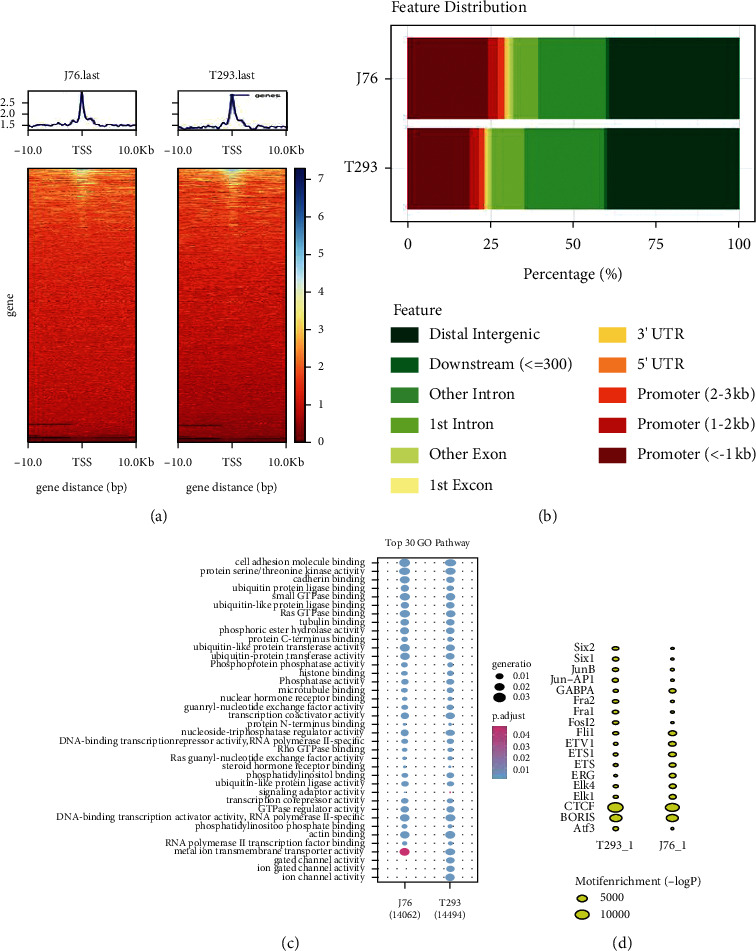
Main procedures and analysis process of ATAC seq in J76 cells and 293T cells. (a) Heatmap analysis of the enrichment of ATAC- seq signal around TSS. (b) Distribution of chromatin open region peaks in gene functional elements. (c) Go enrichment analysis of peak-related genes in the chromatin open region of J76 cells and 293T cells. (d) Comparison of motif analysis results between J76 cells and 293T cells.

The second main step of ATAC-seq data analysis is the element of advanced analysis, which determines the accessible areas (also known as peak value). In order to identify accessible regions, MACS2 was used as the peak caller for ATAC-seq, and the number and average length of peaks were counted to obtain the chromatin open region. It was found that there were 52,224 differentially expressed chromatin open regions and 25,929 differentially expressed chromatin open regions in J76 cells and 293T cells, respectively, and 28,908 coexpressed chromatin open regions. In order to further determine the distribution of chromatin open region expression peaks in the genome, the computed matrix reference point model of deepTools was used to calculate the distribution of peaks in the whole genome before and after the transcription start site (TSS) at 10°K, and a heat map was used to visualize the coverage depth. A plot profile is used to show the coverage depth in the form of a line chart and the analysis results are represented by a heat map, as shown in Figure 3(a). The heat map showed that the ATAC-seq signals of J76 cells and 293T cells were significantly enriched around TSSs. It is suggested that there is an obvious area of chromatin accessibility around TSSs.

### 3.7. Distribution of Peaks in Functional Gene Elements

After achieving the peak sets through the previous procedure, the peaks are annotated by the nearest genes or regulatory elements; the annotation of the peaks can connect chromatin accessibility with gene regulation. The peaks were assigned to overlapping or nearest gene, intron, exon, 5′ untranslated region (UTR), promoter, 3′ UTR, distal intergenic, downstream (< = 300), and other introns and other exon gene regulatory elements (Figure 3(b)). Here, we used R-Pack, ChIPseeker, and ChIPpeakAnno to calculate the characteristics of the two samples. We screened the ATAC-seq signal enhancement regions with *P* value less than 0.05 and changed multiples more than 1.5 times, analyzed the peak annotation, and compared the analysis results of J76 cells and 293T cells, as shown in Figure 3(b). The gene function of the open chromatin region of J76 cells was higher than that of 293T cells (<1kb), but the intron region of J76 cells was lower than that of 293T cells.

### 3.8. GO Enrichment Analysis of Peak Associated Genes

The chromatin open region is often distributed within actively transcribed genes and gene elements with regulatory functions. In order to further determine the distribution of chromatin accessibility, we analyzed the biological function of the open regions in the two kinds of cells. The peak-related genes were calculated by using the R package ChIPseeker, and then GO enrichment analysis was performed for these genes, as shown in Figure 3(c). The difference of gene enrichment associated with the chromatin open region between the two cells is the molecular function, which is mainly reflected in three molecular functions: gate channel activity, ion gate channel activity, and ion channel activity.

### 3.9. Motif Analysis

TFs (transcription factors) affect the transcription of open chromatin, which can promote the transcription by recognition and binding to characteristic sequences on DNA. These sequences are called motifs, and the combined positions are known as TFBS (TF binding sites, TFBS). Therefore, explaining motif function or activity changes may promote deciphering the potential relative networks while, in the meantime, identifying essential regulators. We used Homer to analyze the motif of the sequencing results and obtained the transcription factors that bind to the chromatin open region, showing the top 10 motifs with the most significant enrichment, as shown in Tables 1 and 2. The forecasted TFBSs indirectly from sequences found in the insert peak area. The motif results of the two kinds of cells were compared and analyzed, as shown in Figure 3(d). The ETS (E26 transformation specific sequence) family was the most significant transcription factor in the 293T cell motif analysis, while the AP-1 family was the most significant transcription factor in the J76 cell motif analysis.

## 4. Discussion

Since Buenrostro et al. [10] established ATAC-seq, it has been widely used and has become an important method to study epigenetics by analyzing chromatin accessibility. However, the most efficient ATAC-seq library manufacturing procedure available up to now is due to a robust Tn5 transposase. The homologous wild-type transposable unit exists in bacteria and consists of two almost identical but opposite regions, IS50R and IS50L, yet Tn5 transposase is encoded by IS50R. Tn5 relies on the homodimer combined with the outside end (OE) to finish the shift translocation of the transposable units through a mechanism that was known as “cut-and-paste” [19].

The mutation site of wild-type Tn5 is M56, and the transposition frequency is very low. Through the construction of mutants containing E54K and L372P mutation sites, the Tn5 functional elements are a stable complex composed of two oligonucleotide adapters and two transposase molecules, and the adapters bind to the transposase relying on a double-stranded mosaic end (MES) sequences. The process allows the Tn5 transposase to cut the DNA and connect the adapter sequences to the 5′ends of the fragments.

In the downstream purification process of Tn5 transposase, chitin purification columns are primarily used. This method is complex, takes a long time, and cannot obtain the library repeatedly [14]. Furthermore, the costs and throughput of library preparation need to be improved, which is a serious limiting factor in many ATAC-seq based research projects [20, 21]. Here, the recombinant plasmid pTXB1-Tn5 was constructed and transformed into BL21. After adding polyethyleneimine (PEI) into the crude lysate to remove the DNA, loading the extracted lysate onto a chitin magnetic bead column, and relying on cutting intein–CBD labels was induced by DTT, so Tn5 was released from the column. Finally, the concentration of Tn5 protein was measured by a Nanodrop spectrophotometer. When a new batch of the enzyme is purified, confirmation of its activity by digesting DNA into diffuse bands with 5 ng/ml Tn5. The Tn5 protein needs to be assembled into transposons every time before tagmentation. The actual concentration reached 13.62 ng/ml and was then assembled by using oligonucleotide primers Tn5ME-A, Tn5ME-B, and Tn5Merev synthesized in advance, determining the tagmentation of genomic DNA samples. In brief the results show that the purified Tn5 structures are very successful and synthesize a library fit for sequencing.

In order to establish a complete system of ATAC-seq technology, the representative metabolic system human kidney epithelial cell line 293T and the immune system human peripheral blood leukemia T cell line Jurkat were selected as the test samples, and the Tn5 transposase was used for tagmentation. The sequencing data were analyzed by the systems biology analysis method.

The epigenetic function of the chromatin open region of 293T cells and J76 cells was studied by using ATAC-seq and analyzing methods such as TSS signal enrichment, GO enrichment, and motif analysis. The results showed that the difference in expression peaks in the open region of chromatin between J76 and 293T cells was 52,224 and 25,929, respectively. The peaks of the two cells were enriched around TSSs. The TSS is a region where transcription factors and regulatory elements combine with each other, and the accessibility of chromatin is higher. By obtaining peak sets, the peaks were assigned to the nearest or overlapping gene (Figure 3(b)). It indicated that the remarkable distribution of chromatin open region peaks was in the distal intergenic, promoter, and intron regions of gene functional elements, while the promoter region (<1kb) of J76 cells was higher than 293T cells, and the intron region was significantly lower than 293T cells. The difference in the concentration and analysis of GO is mainly reflected in the activity of ion channels, which is consistent with the main function of the cell. 293T cells are derived from human kidney epithelial cells, and more ion channels play a role. In order to obtain the functional genes of chromatin open regions, transcription factor binding predictions of the two cells were obtained by motif analysis. The TFs related to the motifs of 293T cells were Fli, ETV1, ETS1, ETS, ERG, ELK4, and ELK1. In J76 cells, they were Six2, Six1, junB, jun-ap1, fra2, fra1, Fosl2 and ATS3.

The TFs with high correlation in 293T cell motif analysis belong to the ETS transcription factor family, which is one of the largest transcription regulatory factor families in cells. Most of them are transcriptional activators involved in the regulation of embryonic development, cell growth, differentiation, apoptosis, etc., and regulate many physiological and pathological processes. FLI-1 mainly exists in vascular endothelial cells, macrophages, immune cells, the hematopoietic system, and fibroblasts, and participates in the regulation of inflammatory response, embryonic development, cell proliferation, differentiation, and migration [22]. ETV1 can bind many target genes that play a key role in regulating differentiation, proliferation, and tumor metastasis by regulating the expression of target genes [23, 24]. Ets-1 is highly expressed in B cells and lymphoid organs, to inhibit the differentiation of plasma cells and may also be involved in embryo implantation [25]. ERG is involved in physiological and pathological angiogenesis, regulation of vascular development, control of endothelial differentiation and reprogramming, maintenance of peripheral platelet count, and the normal generation of megakaryocytes. Elk-1 is the substrate of the MAPK cascade, which induces the phosphorylation of Elk-1 [26]. Serum response factor (SRF) and Elk-1 work together to activate the promoter of c-fos [27]. Elk-4 is expressed in hematopoietic or endothelial cells during early vertebrate embryogenesis [28]. The TFs with a high correlation with motif analysis of J76 cells are all leucine transcription factors of the AP-1 family. They regulate cell proliferation, differentiation, and programmed death by forming various dimer complexes. Fra2 is highly expressed in the ovary, stomach, small intestine, large intestine, brain, lung, and heart tissues, and the upper basal layer of skin [28]. Fra-1 is highly expressed in many tumors and transformed cell lines such as breast, colon, and lung, which can promote malignant transformation by inducing cell proliferation and inhibiting apoptosis. JunB combines with the sequence of the targeted gene's cis-regulatory domain, thus regulating a variety of biological processes, including cell proliferation, cycle, and apoptosis and plays a role in promoting or suppressing cancer [28, 29]. Six1 and Six2 are homologous typing transcription regulators, which can affect and regulate the downstream gene expression related to tumor cell migration and proliferation through a variety of signaling pathways [30]. Fosl2 is differentially expressed in many tumors and is essential to cell cycle regulation and tumor proliferation [31, 32].

## 5. Conclusions

In this study, we produced a highly active Tn5 and established a successful tagmentation procedure that can be taken advantage of in ATAC-seq applications. 293T cells and Jurkat cells were used as samples, and the chromatin open region was analyzed efficiently and accurately. The position of nucleosomes was determined, the TF binding sites were predicted, and the functional genomic regulatory region information of chromatin open regions was determined. Furthermore, the ATAC-seq assay established in this paper can be applied to the analysis of genetic differences and similarities between individuals, etiology of complex diseases, clinical classification and individualized medical treatment, etc. At the same time, ATAC-seq is integrated with RNA-seq, CHIP-seq and other high-throughput sequencing technologies to reconstruct the regulatory network [16] and to provide technical support for the diagnosis of major diseases and the study of molecular mechanisms.

## Figures and Tables

**Table 1:** Motif analysis of the chromatin open region in J76 cells.

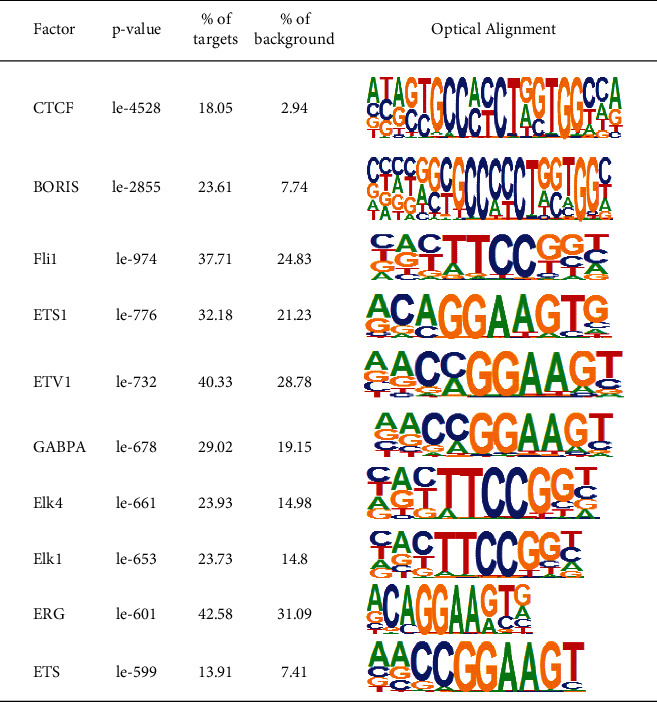

**Table 2:** Motif analysis of chromatin open region in 293T cells.

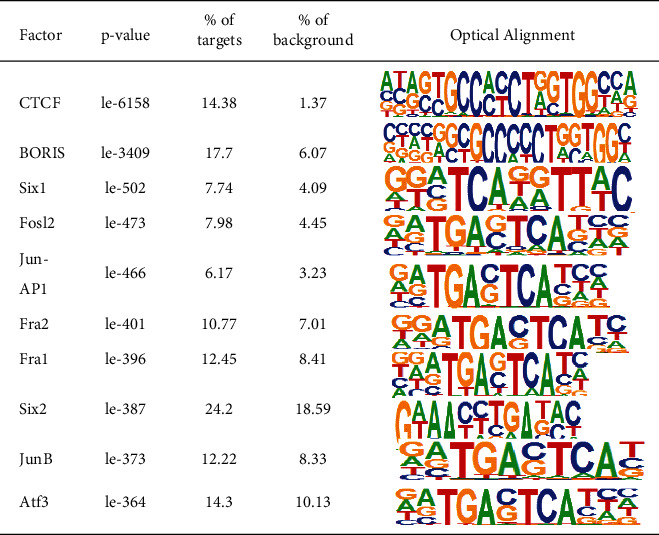

## Data Availability

The data used to support the findings of this study are included within the article. The data that support the findings of this study are openly available in https://www.ncbi.nlm.nih.gov/geo/query/acc.cgi?acc=GSE199016, reference number (FW: GEO Submission (GSE199016)). The data can be viewed on May 1, 2022.
